# Comparing biological networks via graph compression

**DOI:** 10.1186/1752-0509-4-S2-S13

**Published:** 2010-09-13

**Authors:** Morihiro Hayashida, Tatsuya Akutsu

**Affiliations:** 1 Bioinformatics Center, Institute for Chemical Research, Kyoto University,Gokasho, Uji, Kyoto, 611-0011, Japan

## Abstract

**Background:**

Comparison of various kinds of biological data is one of the main problems in bioinformatics and systems biology. Data compression methods have been applied to comparison of large sequence data and protein structure data. Since it is still difficult to compare global structures of large biological networks, it is reasonable to try to apply data compression methods to comparison of biological networks. In existing compression methods, the uniqueness of compression results is not guaranteed because there is some ambiguity in selection of overlapping edges.

**Results:**

This paper proposes novel efficient methods, CompressEdge and CompressVertices, for comparing large biological networks. In the proposed methods, an original network structure is compressed by iteratively contracting identical edges and sets of connected edges. Then, the similarity of two networks is measured by a compression ratio of the concatenated networks. The proposed methods are applied to comparison of metabolic networks of several organisms, *H. sapiens, M. musculus, A. thaliana, D. melanogaster, C. elegans, E. coli, S. cerevisiae,* and *B. subtilis,* and are compared with an existing method. These results suggest that our methods can efficiently measure the similarities between metabolic networks.

**Conclusions:**

Our proposed algorithms, which compress node-labeled networks, are useful for measuring the similarity of large biological networks.

## Background

Development of algorithms for comparing various kinds of biological data is one of the important topics in bioinformatics and systems biology. Methods for comparison of DNA and/or protein sequences have been extensively studied and have been applied to analyses of real sequence data quite successfully. Due to increased interests in systems biology, extensive studies have recently been done on comparison of biological networks.

For comparison of metabolic networks, Ogata et al. developed a method based on clustering [1], Tohsato et al. extended a multiple sequence alignment technique to multiple alignment of metabolic pathways using a scoring scheme based on EC (Enzyme Commission) numbers [2], Pinter et al. applied a tree matching technique to alignment of metabolic pathways [3], and Wernicke and Rasche developed a simple backtracking algorithm utilizing the local diversity property [4]. For comparison of protein-protein interaction networks, Kelley et al. developed PathBlast using dynamic programming [5], Liang et al. developed NetAlign using a clique-based method for computing maximal common subgraphs [6], Li et al. developed MNAligner using integer quadratic programming [7], Singh et al. developed IsoRank algorithm based on Google's PageRank method [8], and Zaslavskiy et al. developed a gradient ascent-based method and a message passing-based method [9].

On the other hand, data compression methods have been applied to comparison of large sequence data [10,11] and protein structure data [12,13]. Since it is still difficult to compare global structures of large biological networks and data compression-based methods can be applied to comparison of large-scale sequence data, it is reasonable to try to apply data compression methods to comparison of biological networks. In this paper, we propose such methods.

In order to apply data compression to biological networks, data compression methods for graphs are required. For compression of graphs, Adler and Mitzenmacher developed a method based of Huffman coding of vertices [14], Peshkin developed GRAPHITOUR based on iterative contractions of identical edges [15], and Cook and Holder developed SUBDUE based on contraction of frequent subgraphs and MDL (minimum description length) principle [16], which was further extended to EDIF for lossless compression by Yang et al. [17]. However, the method by Adler and Mitzenmacher does not seem to be useful for comparison of networks because it does not make much use of structural information. In GRAPHITOUR, the uniqueness of compression results is not guaranteed because there is some ambiguity in selection of overlapping edges (isomorphic graphs may be differently compressed depending on the orderings of vertices in input data), which is not suitable for comparison of network structures. This point is also unclear in EDIF and SUBDUE. Therefore, we develop in this paper novel graph compression methods for which it is guaranteed that two isomorphic graphs are compressed in the same way. Using these compression methods, we measure the similarity of two networks by means of the universal similarity metric (USM) proposed by Li *et al.*[11]. USM is defined using Kolmogorov complexity which represents the amount of information contained in data, and is obtained by removing redundant parts maximally. Therefore, Kolmogorov complexities are approximated by compression sizes.

We apply the proposed methods to comparison of metabolic networks, and compare the results with those of GRAPHITOUR. The results of hierarchical clustering for several organisms suggest that the proposed methods outperforms GRAPHITOUR, and can adequately measure the similarities between metabolic networks.

## Methods

### Graph compression method

Since our proposed methods are based on GRAPHITOUR, we briefly review the GRAPHITOUR algorithm [15]. GRAPHITOUR is based on iterative contractions of identical edges. In order to efficiently contract edges, GRAPHITOUR selects edges appearing most frequently, and solves an instance of *maximum cardinality matching* problem, which finds as many edges as possible such that no two edges share a common vertex.

**Figure 1:**
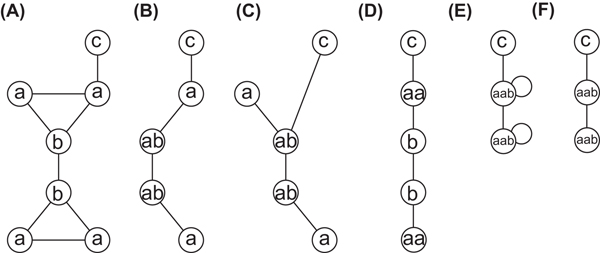
**Example of contraction of identical edges**. (A) Graph with 7 vertices and 8 edges. (B, C) Graphs contracted from graph (A) by GRAPHITOUR. (D) Graph contracted from graph (A) by our proposed method. (E) Graph contracted from graph (A) by substituting two edges ('a', 'a') and ('a', 'b') with 'aab'. (F) Graph contracted from graph (A) by substituting three vertices and the edges between the vertices with 'aab'.

Figure 1 shows an example of contraction of identical edges. The graph of (A) contains 4 edges labeled with 'a' and 'b', 2 edges with 'a' and 'a', 1 edge with 'b' and 'b', and 1 edge with 'a' and 'c'. GRAPHITOUR selects edges with 'a' and 'b' because they appear most frequently, and solves the maximum cardinality matching problem for their edges. However, optimal solutions are not necessarily uniquely determined. (B) shows a contracted graph after the top-left edge with 'a' and 'b' is substituted with a new vertex labeled with 'ab'. On the other hand, (C) shows a contracted graph after the top-right edge with 'a' and 'b' is substituted. This example implies that GRAPHITOUR can generate different compressed graphs. In order to measure the similarity of networks, the same compressed graph should always be obtained. Therefore, we improve GRAPHITOUR for that purpose, and propose the following algorithm, which we call CompressEdge, to uniquely determine contracted edges in each iteration.

Procedure CompressEdge

**Input:** undirected graph *G*(*V*, *E*) with labeled vertices *V* and edges *E* (a total order ≤ is defined for the set of labels *L*, and each *ν* ∈ *V* is labeled with *l_ν_* ∈ *L*); 

**Output:** induced compression rules *R* and compressed graph;

Begin

*R* := ∅;

*s*(*l*) := {*l*} for each label *l* ∈ *L*;

**while** |*E*| > 0

*ε*(*l*_1_, *l*_2_) := {(*ν*_1_, *ν*_2_) ∈ *E*|  = (*l*_1_, *l*_2_) where , *l*_1_ ≥ *l*_2_};

*ε* := {*ε*(*l*_1_, *l*_2_)| no two edges in *ε*(*l*_1_, *l*_2_) share a common vertex};

if *ε* = ∅ then **return** (*R*,*G*);

*ε_most_* := {*ε*(*l*_1_, *l*_2_) ∈ *ε*| |*ε*(*l*_1_, *l*_2_)| ≥ |*ε*(*l*_3_, *l*_4_)| for all *ε*(*l*_3_, *l*_4_) ∈ *ε*};

**select***ε*(*l*_1_, *l*_2_)(∈ *ε_most_*) such that *s*(*l*_1_) ∪ *s*(*l*_2_) <*s*(*l*_3_) ∪ *s*(*l*_4_)

or (*s*(*l*_1_) ∪ *s*(*l*_2_) = *s*(*l*_3_) ∪ s(*l*_4_) and (*l*_1_, *l*_2_) < (*l*_3_, *l*_4_)) for all *ε*(*l*_3_, *l*_4_) ∈ *ε_most_*,

where *l*_1_ ≥ *l*_2_, *l*_3_ ≥ *l*_4_;

**add** a new label *l_n_* to *L* such that *l_n_* >*l* for all *l* ∈ *L*;

*s*(*l_n_*) := s(*l*_1_) ∪ *s*(*l*_2_);

*R* = *R* ∪ {*l_n_* ← (*l*_1_, *l*_2_)};

**for** each edge *e* ∈ *ε*(*l*_1_, *l*_2_)

**substitute***e* with a new vertex labeled with *l_n_*;

**return** (*R*, *G*);

End

This proposed algorithm avoids contraction of edges which share a common vertex. In the example of Figure 1, our algorithm does not choose edges whose endpoints are 'a' and 'b', instead chooses the second candidate edges whose endpoints are 'a' and 'a', and obtains the graph of (D) as the result. It should be noted that the proposed algorithm does not solve the maximum cardinality matching problem because it selects only edges such that all edges with the same labels do not share a common vertex.

However, it is not sufficient to uniquely determine contracted edges because there can be more than one set which has the same number of edges, that is, |*ε_most_*| > 1. Therefore, we introduce a total order to sets of labels to determine the priority of edges. Each edge has a pair of labels (*l*_1_, *l*_2_) corresponding to two endpoints of the edge. Let *s*_1_ and *s*_2_ be sets of labels. We can define a total order for *s*_1_ and *s*_2_ as follows.

First, we sort *s*_1_ and *s*_2_ by descending order, respectively. We compare *i*-th elements  , of *s*_1_ and *s*_2_, and define *s*_1_ <*s*_2_ if  < and  = (for all *j* <*i*) hold for some *i*. The proposed algorithm selects edges with the smallest set of labels from *ε_most_* according to the total order. For example, if we compare *s*_1_ = {*l*_1_, *l*_3_} with *s*_2_ = {*l*_3_, *l*_2_} under *l*_1_ <*l*_2_ <*l*_3_, *s*_1_ and *s*_2_ are sorted as (*l*_3_, *l*_1_) and (*l*_3_, *l*_2_), respectively, and we have *s*_1_ <*s*_2_.

When edges with (*l*_1_, *l*_2_) are contracted, a new label *l_n_* is added to *L*, where *l_n_* >*l* for all *l* (≠ *l_n_*) ∈ *L*. In computational experiments, Morgan index [18] based on graph structures is assigned to each vertex. However, new added labels themselves do not reflect the original graph structure. Therefore, in order to make effective use of the total order of original labels, we introduce a set of labels for each label *l*, *s* (*l*), which consists of only original labels. Then, *s* (*l_n_*) is defined to be *s* (*l*_1_) ∪ *s* (*l*_2_) when (*l*_1_, *l*_2_) is substituted with *l_n_*. The algorithm compares *s* (*l*_1_) ∪ *s*(*l*_2_) with *s* (*l*_3_) ∪ *s* (*l*_4_) before comparing edges of (*l*_1_, *l*_2_) and (*l*_3_, *l*_4_). For example, for the graph of Figure 1D, the algorithm selects edges with ('aa', 'b') as contracted edges because it appears most frequently. However, if there is another edge with ('b', 'b') than shown in Figure 1D, edges of ('aa', 'b') and ('b', 'b') are compared. We suppose that 'a'<'b'<'c'<'aa' and 'aa' was obtained by contracting edges with ('a', 'a'). Then, the corresponding sets to ('aa', 'b') and ('b', 'b'), *s*_1_ = *s*('aa') ∪ *s*('b') = {'a','a','b'} and *s*_2_ = *s*('b') ∪ s('b') = {'b','b'}, are compared, sorted as {'b','a','a'} and {'b','b'}, respectively, and we have *s*_1_ <*s*_2_. Then, edges with ('aa', 'b') are selected, and contracted to vertices with a new label 'aab', where 'aab'>'aa' and *s* ('aab') = *s* ('aa') ∪ *s*('b') = {'a','a','b'}.

### Extension to contraction of multiple edges

In the previous algorithm, identical edges are contracted at each iteration. In this section, we propose another algorithm, which we call CompressVertices, to contract identical sets of multiple connected edges. In order to uniquely determine the contracted sets of connected edges, we must introduce a total order to a set of edges. For that purpose, we apply degree sequence [19], which is defined to be the non-increasing sequence of degrees of vertices. For example, the degree sequence of Figure 1A is (3, 3, 3, 2, 2, 2, 1). Moreover, in order to distinguish labels of vertices, we introduce the non-increasing sequence of pairs of the degree and the label for each vertex included in the set of edges, which we call dl-sequence. In dl-sequence, the degree is not calculated for the original graph, but is for the set of edges, and we define the inequality of elements of dl-sequence by; (*d*_1_, *l*_1_) > (*d*_2_, *l*_2_) if *d*_1_ >*d*_2_ or (*d*_1_ = *d*_2_ and *l*_1_ >*l*_2_). Then, we can define a total order for dl-sequences  and  by and  hold for some *i*. For example, the dl-sequence of two connected edges of ('a', 'a') and ('a', 'b') in Figure 1A, that is a–a–b, is ((2, 'a'), (1, 'b'), (1, 'a')). Here, in the example, if the edges are substituted with a new vertex, a self loop is remained to the new vertex due to the edge of (' a', 'b') as shown in Figure 1E. The remained edges should be also contracted. Therefore, we modify CompressEdge, and propose the following algorithm to contract identical sets of vertices and the edges between the vertices instead of individual edges. In the example of Figure 1E, the graph of Figure 1F is obtained by this algorithm.

**Procedure CompressVertices***(M)*

**Input:** maximum number of vertices *M* and undirected graph *G*(*V*, *E*) with labeled vertices *V* and edges *E*

**Output:** induced compression rules *R* and compressed graph;

Begin

*R* := ∅;

*s*(*l*) := {*l*} for each label *l* ∈ *L*;

**while** |*E*| > 0

*V* = ∅; *m* := 2;

**while***V* = ∅ and *m* ≤ *M*

(*V*, *dl_i_*) := SelectVertices(*m*);

*m* := *m* + 1;

if *V* = ∅ then **return** (*R*, *G*);

**add** a new label *l_n_* to *L* such that *l_n_* >*l* for all *l* ∈ *L*;

*R* = *R* ∪ {*l_n_* ← *dl_i_*};

**for** each set of vertices *u* ∈ *V*

**substitute***u* with a new vertex labeled with *l_n_*;

**return** (*R*, *G*);

End

**Subprocedure SelectVertices**(*m*)

Begin

*V*(*dl_i_* := ((*d*_1_, *l*_1_), …, (*d_m_*, *l_m_*))) := {{*ν*_1_, …, *ν_m_*} ⊂ *V* | *ν*_1_, …, *ν_m_* are connected and (the dl-sequence of {*ν*_1_, …, *v_m_*})= *dl_i_*};

*V* := {*V*(*dl_i_*)| *u*_1_ ∩ *u*_2_ = ∅ for all *u*_1_,*u*_2_ ∈ *V*(*dl_i_*)};

if *V* = ∅ then **return** (∅, ∅);

*V_most_* := {*V*(*dl_i_*) ∈ *V*| |*V*(*dl_i_*)| ≥ |*V*(*dl_j_*)| for all *V*(*dl_j_*) ∈ *V*};

**select***V*(*dl_i_*)(∈ *V_most_*) such that 

or (*s_i_* = *s_j_* and *dl_i_* <*dl_j_*) for all *V*(*dl_j_*) ∈ *V_most_*;

**return** (*V*(*dl_i_*),*dl_i_*);

End

**Figure 2:**
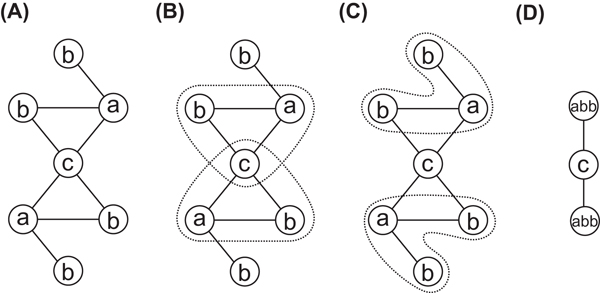
**Example of contraction by CompressVertices** Example of contraction of identical sets of vertices and the edges between the vertices by CompressVertices. (A) Graph with 7 vertices and 8 edges. (B) Candidate vertices {' a', 'b', 'c '} and three edges ('a', 'b'), ('b', 'c'), and ('c', 'a') to be contracted. (C) Candidate vertices {'a', 'b', 'b'} and two edges of ('a', 'b') to be contracted. (D) Graph contracted from graph (A) by substituting vertices {'a', 'b', 'b'} and the edges with 'abb'.

First, the algorithm tries to find two vertices and the edge between the vertices to be contracted. If it does not find such two vertices, it tries to find more than two vertices and the edges between the vertices until it finds or up to the given number of vertices, *M*. Figure 2 shows an example of contraction by the algorithm. The graph of (A) contains 4 edges labeled with 'a' and 'b', 2 edges with 'a' and 'c', and 2 edges with 'b' and 'c'. However, it cannot select any edge because they are overlapping each other. Therefore, it tries to find three vertices to be contracted. (B) shows the candidate sets of vertices {' a', 'b', 'c '} and three edges ('a', 'b'), ('b', 'c'), and ('c', 'a'), which appear most frequently two times in the graph. However, they are overlapping again. Finally, it selects the candidate sets of (C), vertices {'a', 'b', 'b'} and two edges of ('a', 'b'), which appear also most frequently. (D) shows the graph contracted from the graph (A) by substituting the selected vertices and edges with a new vertex labeled with 'abb'.

CompressVertices(2) of *M* = 2 is equivalent to CompressEdge. It should be noted that the algorithm uniquely determines the contracted sets of connected edges in each iteration although different subgraphs can be substituted with vertices of the same label in *M* ≥ 4. For example, both graphs of a-b-a-b and a-a-b-b are represented as ((2, 'b'), (2, 'a'), (1, 'b'), (1, 'a')) in dl-sequence. It is to be noted that for three vertices, different subgraphs cannot have the same dl-sequence. The algorithm is still efficient because it compares dl-sequences instead of comparing subgraphs, where subgraph isomorphism problem is known to be in NP-complete.

### Similarity measure

The universal similarity metric (USM) was proposed by Li* et al.*[11], and has been applied to several biological data [12,13]. USM between two objects *o*_1_ and *o*_2_ is defined using Kolmogorov complexity *K*(*o*) as follows:

(1)

where  denote shortest programs for generating *o*_1_,*o*_2_, respectively.

Kolmogorov complexity *K*(*o*) of an object o is defined to be the length of the shortest program *P* for a universal Turing machine *U* which outputs *o*, and the conditional Kolmogorov complexity of *o*_1_ given *o*_2_ is defined to be the length of the shortest program *P* which outputs *o*_1_ when *o*_2_ is given as follows:

(2)

It should be noted that *K*(*o*) is considered as a measure of the amount of information that the object *o* contains.

Since it is not possible to obtain these Kolmogorov complexities for real data, we approximate *K*(*G*) of a graph *G* by *C*(*G*) = |*R*| + |*E_C_*|, where |*R*| means the number of rules extracted from *G* by our method, and |*E_C_*| means the number of remaining edges after the compression of *G*. The conditional Kolmogorov complexity *K*(*G*_1_|*G*_2_) of *G*_1_ given *G*_2_ can be approximated to be *C*(*G*_1_ ∪ *G*_2_) − *C*(*G*_2_) as in [12,13], where *G*_1_ ∪ *G*_2_ means the concatenated graph *G*′(*V*′, *E*′) of *G*_1_(*V*_1_, *E*_1_) and *G*_2_(*V*_2_, *E*_2_) such that *V*′ = *V*_1_ ∪ *V*_2_, *E*′ = *E*_1_ ∪ *E*_2_, |*V*′| = |*V*_1_| + |*V*_2_| and |*E*′| = |*E*_1_| + |*E*_2_|. Even if there are identical vertices (i.e. vertices with identical labels) between *G*_1_ and *G*_2_, they are added to *V*′ as different vertices.

Substituting *K*(*o*) of Eq.(1) with *C*(*G*), the approximated USM for graph compression, GUSM, between two graphs *G*_1_ and *G*_2_ is given as follows:

 (3)

It should be noted that *GU SM*(*G*, *G*) = 0 if |*E_c_*| =0 for *C*(*G*). If *G*_1_ and *G*_2_ are similar, then *G*_1_ and *G*_2_ are generated from almost the same set of rules *R*, that is, *C*(*G*_1_ ∪ *G*_2_) ≈ *C*(*G*_1_) ≈ *C*(*G*_2_) ≈ |*R*|, and *GU SM*(*G*_1_ ,*G*_2_) approaches to 0.

## Results and discussion

To evaluate the proposed measure, we used metabolic pathways for several organisms, *H. sapiens, M. musculus, A. thaliana, D. melanogaster, C. elegans, E. coli, S. cerevisiae,* and *B. subtilis,* from the KEGG database [20] (see Table 1). We used chemical compounds as nodes and chemical reactions as edges. For evaluation purposes, it is not appropriate to use protein-protein interaction networks because the accuracy of the networks is not sufficient [21]. Furthermore, we compared the results with those of GRAPHITOUR because other methods such as PathBLAST [5], NetAlign [6], and MNAligner [7] do not give the similarity of networks, and focus on finding interesting subnetworks or most similar subnetworks to a query network.

**Table 1:** Statistics of metabolic pathways

organism	# nodes	# edges
*H. sapiens*	1550	1673
*M. musculus*	1518	1640
*A. thaliana*	1389	1395
*D. melanogaster*	1238	1250
*C. elegans*	1049	1009
*E. coli*	1103	1256
*S. cerevisiae*	983	1028
*B. subtilis*	994	1065

Statistics of metabolic pathways for several organisms, *H. sapiens, M. musculus, A. thaliana, D. melanogaster, C. elegans, E. coli, S. cerevisiae,* and *B. subtilis.*

In our first computational experiment, all nodes in the metabolic networks were labeled with chemical compounds, and there was only one edge having the same labels, that is, |*ε*(*l*_1_, *l*_2_)| = 1. Then, our compression algorithm for *G*(*V*, *E*) produced rules *R* and the remaining graph *G_c_*(*V_c_*, *E_c_*) as *C*(*G*) = |*R*| + |*E_c_*| = |*E*|. This means that *G* is not compressed. Many other methods also cannot select frequent subnetworks for such networks.

**Figure 3:**
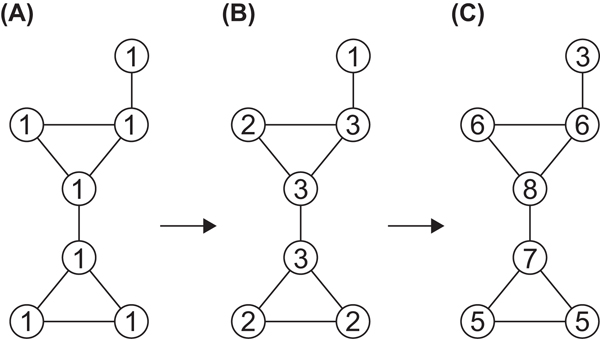
**Example of calculation of Morgan index**. (A) 1 is assigned to each vertex. (B) first iteration. (C) second iteration.

Since we would like to compare network structures for the metabolic networks, we replaced labels with Morgan index [18]. Figure 3 shows an example of calculation of Morgan index. First, 1 is assigned to each node. Next, the sum of values of adjacent nodes is assigned for each node. This iteration is repeated until the number of different values of Morgan index does not increase. We call the index obtained in this way the original Morgan index. Morgan index obtained by one iteration of this procedure is equivalent to the degree of each node, and Morgan index depends on graph structures.

We fixed the number of iterations of the Morgan index procedure, applied our compression algorithms to individual and concatenated metabolic networks, *G*_1_, *G*_2_, *G*_1_ ∪ *G*_2_, and calculated *GU SM *(*G*_1_, *G*_2_) from *C*(*G*_1_), *C*(*G*_2_) and *C*(*G*_1_ ∪ *G*_2_). To confirm that our compression algorithms work for measuring the similarity of metabolic networks, we obtained hierarchical clustering results using the nearest neighbor (single linkage) method, and compared them with actual phylogenetic trees and hierarchical clustering results by GRAPHITOUR. Moreover, we performed such experiments with several numbers of iterations from 1 to 20 because the number of iterations of the original Morgan index is at most 11 for the metabolic networks.

**Figure 4:**
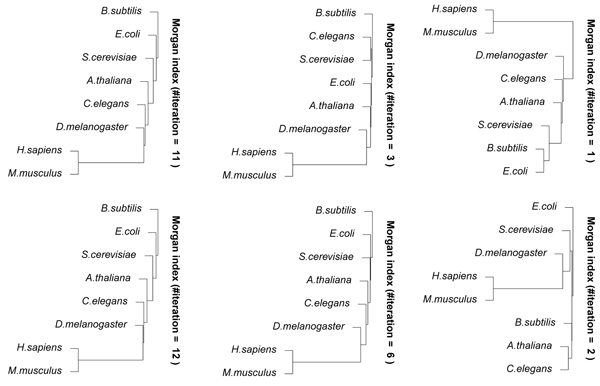
**Results of hierarchical clustering using CompressEdge** for metabolic networks of several organisms, *H. sapiens, M. musculus, A. thaliana, D. melanogaster, C. elegans, E. coli, S. cerevisiae,* and *B. subtilis *with Morgan indices of 1, 2, 3, 6, 11, and 12 iterations.

**Figure 5:**
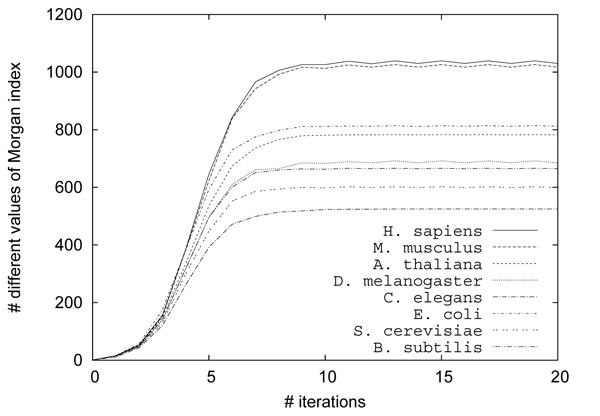
**Results on the number of different values of Morgan indices** for metabolic networks of several organisms, *H. sapiens, M. musculus, A. thaliana, D. melanogaster, C. elegans, E. coli, S. cerevisiae,* and *B. subtilis* for 1-20 iterations of the Morgan index procedure.

Figure 4 shows the results of hierarchical clustering using CompressEdge for metabolic networks of several organisms, *H. sapiens, M. musculus, A. thaliana, D. melanogaster, C. elegans, E. coli, S. cerevisiae,* and *B. subtilis* with Morgan indices of 1, 2, 3, 6, 11, and 12 iterations. The numbers of contracted edges for the metabolic network of H. sapiens with Morgan indices of 1, 2, 3, 6, 11, and 12 iterations were 251, 1367, 1387, 1395, 1395, and 1395, respectively. The results of more than 5 iterations were almost similar to those of 12 iterations. Figure 5 shows the results on the number of different values of Morgan indices for the metabolic networks for 1-20 iterations of the Morgan index procedure. We can see from this figure that the number of different values of Morgan indices is almost constant for more than 11 iterations. For a small number of iterations, it is considered that metabolic networks were not compressed well because many edges have the same labels and share common nodes. This means that the number of iterations is required to be large for measuring the similarity more accurately. However, for that purpose, much larger numbers than the number of iterations of the original Morgan indices, that is at most 11, are not needed because the number of different values of Morgan indices is almost constant for more than 11 iterations (see Figure 5). According to the results of hierarchical clustering in Figure 4, *H. sapiens* was always the nearest to *M. musculus.* Bacterial organisms of *B. subtilis* and *E. coli* were furthest from *H. sapiens* in the result of 12 iterations. It is considered that the result of 12 iterations is almost consistent to actual phylogenetic trees. This suggests that the proposed method can adequately measure the similarities between metabolic networks.

**Figure 6:**
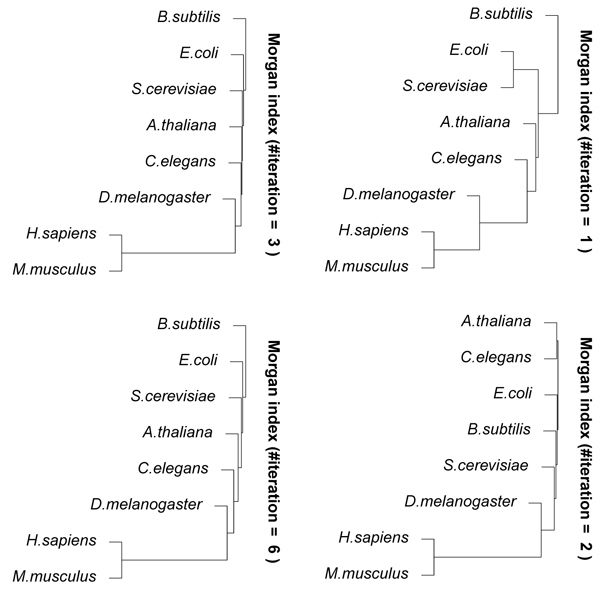
**Results of hierarchical clustering using CompressVertices(3)** for metabolic networks of several organisms, *H. sapiens, M. musculus, A. thaliana, D. melanogaster, C. elegans, E. coli, S. cerevisiae,* and *B. subtilis* with Morgan indices of 1, 2, 3, and 6 iterations.

Figure 6 shows the results of hierarchical clustering using CompressVertices(3) for metabolic networks. The result of 3 iterations was already similar to the results of more than 5 iterations. It is considered that there are more overlapping edges in networks for a smaller number of iterations, and our proposed method contracted their edges well for a small number of iterations.

**Figure 7:**
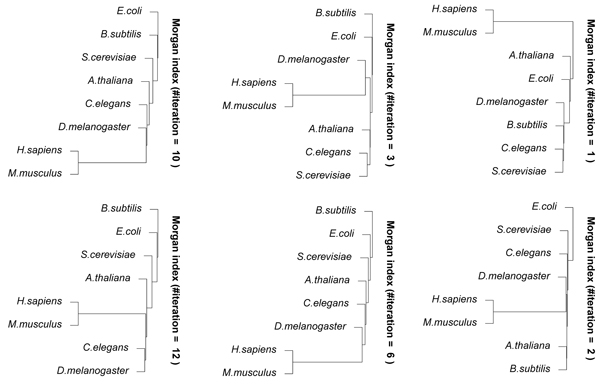
**Results of hierarchical clustering using GRAPHITOUR** for metabolic networks of several organisms, *H. sapiens, M. musculus, A. thaliana, D. melanogaster, C. elegans, E. coli, S. cerevisiae,* and* B. subtilis* with Morgan indices of 1, 2, 3, 6, 10, and 12 iterations.

Figure 7 shows the results of hierarchical clustering using GRAPHITOUR for metabolic networks. In the result of 10 iterations, *E. coli* was farthest from *H. sapiens*. The result of 12 iterations was not well clustered because *D. melanogaster* is likely to be closer to *H. sapiens* than to *C. elegans *[22]. These suggest that our proposed method can measure the similarities better than GRAPHITOUR.

Furthermore, the proposed methods are efficient. The computational time was at most 9 seconds in CompressEdge, 17 seconds in CompressVertices(3), even for the concatenated network of *H. sapiens* and *M. musculus* with Morgan index of 12 iterations. These experiments were done in a single processor core on a PC with Xeon X5460 3.16GHz CPUs and 8GB memory under the Linux (version 2.6) operating system, where the g++ compiler was used with optimization option -O3.

We also compared our proposed methods with simple methods based on node and edge numbers. We calculated distances between two organisms as the differences of the number of nodes or edges, and obtained hierarchical clustering results using the nearest neighbor method. We can see from the results (Figures S1 and S2 in the supplementary information web page) that *H. sapiens* was the nearest to *M. musculus*, but other relations were different from actual phylogenetic trees. This result shows that our proposed methods are better than those based on node or edge numbers. In addition, our methods generate rules to contract edges iteratively. It means that they try to find hierarchical substructures for a given graph. We defined the similarity between graphs, GUSM, based on the number of such rules. It can be considered that GUSM implies whether hierarchical substructures are also similar, or not.

We proposed two methods, CompressEdge and CompressVertices(*M*), where *M* denotes the maximum number of vertices to be contracted at a time. It should be noted that the minimum number is 2 because a vertex itself cannot be contracted. CompressEdge is equivalent to CompressVertices(2). If a graph has many overlapping edges, CompressEdge (and CompressVertices(2)) would not extract many rules, that is, many edges are not contracted and are remained. Consider a graph *G* that all edges of *G* are overlapping. The similarity between *G* and itself, *GU SM *(*G*, *G*), must be 0. However, *GU SM *(*G*, *G*) = 1 because any edge of *G* is not contracted, *C *(*G*) = |*E_c_*| and *C *(*G* ∪ *G*) = 2|*E_c_*|. Therefore, CompressVertices(*M*) for *M* > 2 is needed.

## Conclusions

In this paper, we have proposed novel methods for compressing biological networks. One of the important properties of the proposed methods is that isomorphic networks are compressed in the same way. Unlike many other methods comparing biological networks, our methods are able to give the similarity metric of their networks. Moreover, our methods are very efficient because they do not compare subgraphs directly. We have applied the proposed compression methods to comparison of metabolic networks, and compared the results with those of an existing method. The results suggest that the proposed compression methods are useful for comparison of biological networks.

Our proposed methods were applied to only undirected graphs in this paper. However, it is possible to extend our algorithms to deal with directed graphs. It is easy to extend CompressEdge, and the modified method can still be efficient. Our methods are efficient as well as GRAPHITOUR, and it is important to keep the efficiency after extending them. Metabolic networks can also be represented as directed graphs and undirected graphs using chemical compounds as nodes and the relation between compounds (e.g., involve the same reaction) as edges. It is an interesting issue to examine whether our methods are robust for any representation of a network.

It is considered in general that random networks are more difficult to be compressed than scale-free networks. However, our methods cannot compress metabolic networks that are known as scale-free networks because all nodes in metabolic networks are labeled with distinct chemical compounds, and there is only one edge having the same labels. Our proposed methods are useful only for comparison of networks. If the same labels can appear multiple times, it is expected that our methods can also differentiate these networks. However, it is difficult to compare them in a simple way because the compression size depends on the distribution of node labels. Since we do not have realistic models for generation of random and scale-free networks with node labels, application of our proposed methods to differentiation of random networks from scale-free networks is left as future work.

Though we have applied the methods to comparison of networks, the application is not limited to comparison. It might be applied to detection of network motifs with hierarchical structures because our method iteratively compresses edges (edges can be replaced by small subgraphs).

One drawback of our proposed compression methods is that it is not a lossless compression method (i.e., the original network cannot be reconstructed from compressed data). Therefore, improvement of the method towards lossless compression is also important future work.

## Availability

The source code of CompareVertices is available through the supplementary information web page (http://sunflower.kuicr.kyoto-u.ac.jp/morihiro/gracomp/).

## Competing interests

The authors declare that they have no competing interests.

## Authors' contributions

Methods were developed by MH and TA. The manuscript was prepared by MH and TA. 
